# Viral infection reveals hidden sharing of TCR CDR3 sequences between individuals

**DOI:** 10.3389/fimmu.2023.1199064

**Published:** 2023-05-30

**Authors:** Michal Mark, Shlomit Reich-Zeliger, Erez Greenstein, Adi Biram, Benny Chain, Nir Friedman, Asaf Madi

**Affiliations:** ^1^ Department of Immunology, Weizmann Institute of Science, Rehovot, Israel; ^2^ Division of Infection and Immunity, Department of Computer Science, University College London, London, United Kingdom; ^3^ Department of Pathology, Tel-Aviv University, Tel-Aviv, Israel

**Keywords:** TCR - T cell receptor, LCMV (lymphocytic choriomeningitis virus), epitope-specific T cell, effector T cells, severe acute respiratory syndrome coronavirus 2 (SARS-CoV-2)

## Abstract

The T cell receptor is generated by a process of random and imprecise somatic recombination. The number of possible T cell receptors which this process can produce is enormous, greatly exceeding the number of T cells in an individual. Thus, the likelihood of identical TCRs being observed in multiple individuals (public TCRs) might be expected to be very low. Nevertheless such public TCRs have often been reported. In this study we explore the extent of TCR publicity in the context of acute resolving Lymphocytic choriomeningitis virus (LCMV) infection in mice. We show that the repertoire of effector T cells following LCMV infection contains a population of highly shared TCR sequences. This subset of TCRs has a distribution of naive precursor frequencies, generation probabilities, and physico-chemical CDR3 properties which lie between those of classic public TCRs, which are observed in uninfected repertoires, and the dominant private TCR repertoire. We have named this set of sequences “hidden public” TCRs, since they are only revealed following infection. A similar repertoire of hidden public TCRs can be observed in humans after a first exposure to SARS-COV-2. The presence of hidden public TCRs which rapidly expand following viral infection may therefore be a general feature of adaptive immunity, identifying an additional level of inter-individual sharing in the TCR repertoire which may form an important component of the effector and memory response.

## 1 Introduction

T cell receptor (TCR) antigen recognition is a key step in cellular immunity. The ability to recognize a wide range of different pathogens depends on the huge αβ TCR repertoire diversity generated by the stochastic and imprecise recombination of variable, diversity and joining (VDJ) genes (1). The estimated number of possible TCRs which could be generated has been estimated as greater than 10^14^ (2), exceeding by many orders of magnitude the number of T cells in the human body. Nevertheless, TCR sequences shared between many individuals, often referred to as public TCRs, have been reported in both human (3, 4); and mouse (5, 6). Although some public sequences have been annotated as specific to viral or bacterial antigens (7, 8), most studies have focused on repertoires from healthy individuals, and less is known about the balance between public and private TCRs in the context of acute infection.

Lymphocytic choriomeningitis virus (LCMV) offers an excellent and well-described model in which to study the TCR repertoire associated with acute infection. The Armstrong strain of LCMV is cleared by eight days post-infection, which corresponds to a strong expansion of CD4+ and CD8+ virus-specific T cells (9, 10). This is followed by a contraction phase, giving rise to a subset of long-lived memory T cells maintained by antigen-independent homeostatic proliferation (11). CD4+ memory T cells subsequently decline slowly, while the CD8+ memory population remains relatively stable (12). The magnitude of the CD8+ response is greater than the CD4+ response throughout the response (13). However, CD4+ T cells are essential for an optimum CD8+ memory response. For example, the TCR signal strength of anti-viral CD4+ LCMV specific T cells has been shown to be critical to memory differentiation during the primary response (14, 15).

In C57BL/6 mice infected with LCMV, both CD4+ and CD8+ T cell epitopes have been identified. These epitopes are derived from the viral glycoprotein (GP) or nucleoprotein (NP). Some regions of the viral antigens can stimulate both CD4+ and CD8+ T cells. For example, the GP 66-77 region is dually restricted by both MHC class I and II molecules (16). The immunodominance hierarchy of the epitopes has been characterized in some detail. At the peak of infection, the CD8+ T cell response is dominated by cells that recognize NP396-404, a peptide that binds with high affinity with both H-2Db and H-2Kb (17, 18), followed by the intermediate epitopes NP205-212 and GP92-101 (19).

In this study, we combine antigen-specific tetramer sorting with bulk TCR sequencing of different phenotypic populations of T cells to characterize the T cell receptor (TCR) repertoire at different phases of the LCMV response. We demonstrate that LCMV infection drives a convergent CD8+ effector response across mice, resulting in the detection of emerging shared (public) TCR CDR3 sequences whose publicity cannot be observed in the unimmunized repertoire. A similar phenomenon of emerging public CDR3s was observed in humans infected with SARS-COV-2. These “hidden” public TCRs reveal an under-appreciated level of constraint on the naive TCR repertoire, with important consequences for our understanding of the interaction between the T cell repertoire and viral infection.

## 2 Materials and methods

### 2.1 Animals

Female C57BL/6 mice at five weeks old (Envigo) were injected intravenously with 2X10^5^ PFU of the Armstrong LCMV strain (20). Mice were collected after 8 or 40 days of infection. Healthy control mice were injected with PBS and collected eight days post-treatment. All animals were handled according to regulations formulated by The Weizmann Institute’s Animal Care and Use Committee and maintained in a pathogen-free environment.

### 2.2 SARS-COV-2 consortium and study design

We undertook a case control study nested within our COVID consortium healthcare worker cohort. Participant screening, study design, sample collection, and sample processing have been described in detail previously (21). Briefly, healthcare workers were recruited (between 23^rd^ and 31^st^ March 2020) and underwent weekly evaluation using a questionnaire and biological sample collection for up to 16 weeks when fit to attend work at each visit, with further follow up samples collected at 6 months.

Participants with available blood RNA samples who had PCR-confirmed SARS-COV-2 infection (Roche cobas^®^ diagnostic test platform) at any time point were included. A subset of consecutively recruited participants without evidence of SARS-COV-2 infection on nasopharyngeal swabs and who remained seronegative by both Euroimmun anti S1 spike protein and Roche anti-nucleocapsid protein throughout follow-up were included as uninfected controls.

### 2.3 Sample preparation and T cell isolation

Spleens were dissociated with a syringe plunger, and single-cell suspensions were treated with ammonium-chloride potassium lysis buffer to remove erythrocytes.

Bone marrow cells were extracted from mice femur and tibia bones and were purified with CD3+ T isolated kit (CD3ϵ MicroBead Kit, mouse, 130-094-973, Miltenyi Biotec). Splenic CD4+ and CD8+ cells were purified in two steps: (1) Selection of CD4+ cells (CD4+ T Cell Isolation Kit, mouse, 130-104-454, Miltenyi) (2) Unbound cells were purified for CD8+ cells (CD8a+ T Cell Isolation Kit, mouse, 130-104-07, Miltenyi Biotec). For the tetramers binding reaction, we pooled splenocytes from previously vaccinated mice (5 mice after 8 days post infection) and purified their T cells using the untouched isolation kit (Pan T Cell Isolation Kit II, mouse, 130-095-130, Miltenyi Biotec).

### 2.4 Flow cytometry analysis and cell sorting

The following fluorochrome-labeled mouse antibodies were used according to the manufacturers’ protocols: PB or Percp/cy5.5 anti -CD4, PB or PreCP/cy5.5 anti- CD8, PE or PE/cy7 anti- CD3, APC anti-CD62L, Fitc or PE/cy7 anti- CD44 (Biolegend). Cells were sorted on a SORP-FACS-AriaII and analyzed using FACSDiva (BD Biosciences) and FlowJo (Tree Star) software. Sorted cells were centrifuged (450g for 10 minutes) before RNA extraction.

### 2.5 LCMV -tetramers staining and Flow cytometry sorting

Four monomers (NIH Tetramer Core Facility) with different LCMV epitopes were used: MHCII -GP66–77(H-2Bb), MHCI- NP396-404(H-2Db), MHCI- NP205-212(H-2Kb), MHCI- GP92-101 (H-2Db). Tetramers were constructed via binding Biotinylated monomers to PE/APC – conjugated- streptavidin (according to the NIH protocol). Purified T cells were stained with FITC anti-CD4+ and PB anti-CD8+ and followed by tetramers staining (two tetramers together), for 30 min at room temperature (0.6ug/ml). CD4+ and CD8+ epitope-specific cells were sorted from single-positive gates for one type of tetramer. Using two tetramers together for staining provided a control for nonspecific binding, in addition to using cells collected from the unbinding population (**SI Figure 1B**).

### 2.6 Library preparation for TCR-sequencing

All libraries in this work were prepared according to the published method (22), with minor adaptations for mice and an in-house pipeline for pre-processing of the data. The pipeline introduces unique molecular identifiers attached to individual cDNA molecules, which allows correction for sequencing error PCR bias, and provides a quantitative and reproducible method of library preparation. Full details pre-processing pipeline are published (23).

We used sequences that were fully annotated (both V and J segments assigned), in-frame (i.e., they encode for a functional peptide without stop codons), and with copy number greater than one.

### 2.7 Analysis

All statistical analysis was performed using R Statistical Software (version 4.0.0). The Cosine similarity was computed with the package “coop” (version 0.6-3) (24). With the Olga tool (25) we computed the generation probability for each CDR3βAA sequence.

T cell repertoires were sub-sampled for equal size (n=1000 CDR3AAβ clones in spleen). CDR nucleotide sequences were replicated according to the UMI count number, and then randomly sampled. The average Renyi scores for each k (k = 0, 0.25, 0.5, 1, 2, 4) were calculated from 30 repeats of this random sampling.

The package “vegan” (version 2.5-7) (26) was used to project the Nonmetric Multidimensional Scaling (27) Epitope-specific TCRs were filtered based on: 1) top 1,000 sequences, and 2) absence in the unbinding-tetramer populations and across multiple epitope-specific types. Only the filtered TCRs were annotated to the to the bulk samples (**SI Table 2**).

The five amino acid motifs were computed for each CDR3AA by locating the center base and driving from it two additional amino acids from each direction. The amino acids motif sequences logo and charge were calculated with the packages “ggseqlogo” (28) and “Peptides” (29), respectively.

The probability of generation (pGen) for each CDR3AA β chain was commuted using the Olga package (25). The convergent recombination was inferred by counting the number of CDR nucleotide sequences matched V and J segments for each CDR3AA sequence.

SARS-COV-2 expanded TCRs were defined as any TCR which changed significantly between any two time points. The significance boundaries were defined as the maximum TCR abundance which might be observed at time 2, given its abundance at time 1, given Poisson distribution of counts with p < 0.0001, to give a false discovery rate of <1 in 1000. TCR abundances are normalized for the total number of TCRs sequenced in each sample and expressed as counts/million. From these maximal values at any time point, we calculated the expanded TCRβ frequency.

### 2.8 Data availability

All DNA sequences from young and adult mice have been submitted to the Sequence Read Archive under the identifier PRJNA954849. https://www.ncbi.nlm.nih.gov/sra/PRJNA954849.

## 3 Results

### 3.1 LCMV infection promotes clonal expansion within the CD8+ and CD4 + effector and CD8+ central memory repertoire

**Figure 1:**
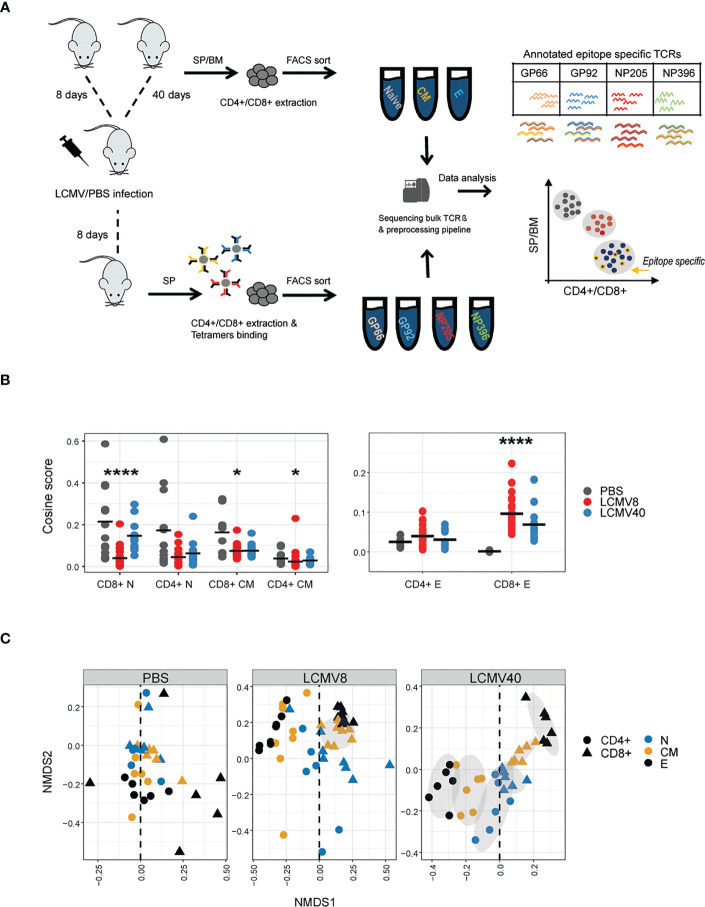
LCMV infection promotes organized TCRβ clonal structure of T cell states, mainly in the expanded CD4+ and CD8+ compartments. **(A)** The experimental design: immunizations, T cell isolation, and TCR repertoire sequencing and analysis pipeline. **(B)** T cell effector repertoires increased clonal similarity during LCMV Infection. Cosine similarity between CDR3AAβ across tissues and mice in each T cell state (effector, central memory and naive) and condition (healthy vs. mice after 8- or 40-days post infection). Horizontal black lines show the mean. Significant differences between mice and tissues are denoted in asterisks (p-values: * < 0.01, ****<0.0001 Kruskal-Wallis test, fdr corrections). **(C)** Non-metric multidimensional scaling (NMDS) representation of similarity between repertoires of different compartments. Each dot represents a T cell state (effector, central memory, and naive in black, orange, and blue, respectively), class (CD4+ in circle, CD8+ in triangle) from a single healthy or LCMV-infected mouse. CDR3AAβs distances between mice, tissues, and compartments were calculated using the cosine similarity index and projected on a plane using NDMS. The grey ellipses on the NDMS panel were computed using the normal confidence ellipses.

We sequenced the TCR repertoire of naive, central memory and effector memory CD4+ and CD8+ T cells from the spleen and bone marrow of three to four C57BL/6 mice at 8 - and 40-days post LCMV infection (summarized in **Figure 1A**). The library preparation incorporates molecular identifiers (UMI) for each cDNA molecule, which allows subsequent correction for PCR bias and sequencing error, allowing a robust and quantitative annotation of each sequence in terms of CDR3 sequence and frequency (22, 23, 30); About ~1.89 x10^6^ annotated CDR3 nucleotide beta chains were obtained, including a varied number of sequences between compartments, tissues, and infection status (**SI Table 1**), which positively correlates with the number of sorted cells (**SI Figure 1C**). Our analysis focuses mainly on the amino acid sequence of the TCR beta complementarity determining region 3 (CDR3βAA), which is the most diverse region of the TCR molecule and is associated with antigen epitope recognition (1).

The abundance distribution profile of the repertoires showed the presence of highly expanded TCRs in the spleen of both CD8+ and CD4+ effector, and in CD8+ central memory T cells 8 days following infection (9, 10). After 40 days of infection, clonal expansion could still be observed in the CD4+ effector, but not the CD8 central memory populations (**SI Figure 1D**). Clonal expansion following infection can also be captured more quantitatively by the set of Renyi diversities, which are shown in **Supplementary Figure 1E**.

Overall, the changes in TCR repertoire in memory and effector populations reflect the known rapid proliferative expansion of memory and effector T cells following infection, providing confidence in the quantitative output of the TCR sequencing pipeline.

### 3.2 Increased CDR3βAA sharing following LCMV infection

We were interested in the impact of infection on driving convergence (increased sharing) versus divergence (decreased TCR sharing) between repertoires. In order to quantify repertoire overlap, while incorporating TCR abundance, we used the pairwise cosine distance between the abundance vectors for each repertoire (see M&M in (23)) to create a matrix of similarities between all pairs of repertoires. We have previously shown that this measure is highly correlated to the Morisita overlap index. LCMV infection drives increased similarity (i.e. increased overlap) within CD4+ and CD8+ effector repertoires 8 days post-infection (peak response), which decreases towards baseline by day 40 (**Figure 1B** -right). No such effect was observed in naive or memory populations (**Figure 1B** -left). An alternative way to visualize the overall pairwise similarity matrix between all the repertoires is to display the matrix in two-dimensional space using multi-dimensional scaling (**Figure 1C**). While the PBS immunized mice show a disordered pattern, dominated by highly divergent effector distributions (perhaps reflecting the heterogeneous previous immunological history of each mouse), infection drove a strong pattern of repertoire convergence, with tight segregation between CD4+ and CD8+ repertoires, tightly clustered effector populations furthest away from naive populations and memory populations in between naive and effectors. This overall pattern was maintained at 40 days post-infection, reflecting long-term stable changes to the repertoire organization following infection.

**Figure 2:**
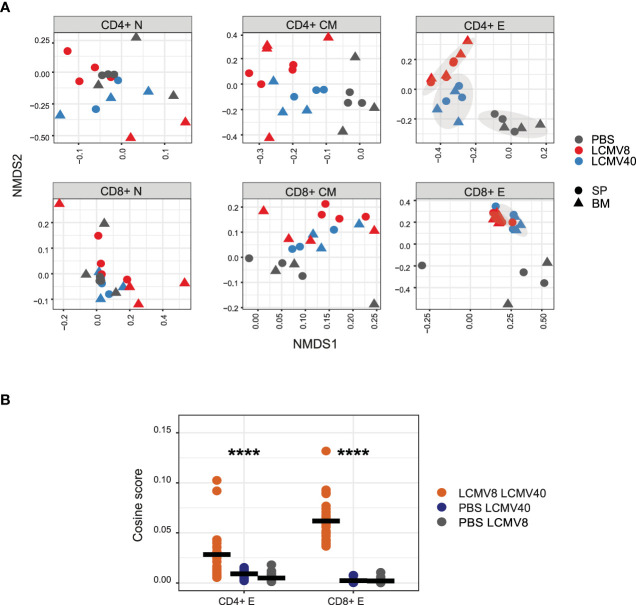
LCMV infection induces common long-lasting T cell effector clones. **(A)** Clonal similarity evaluation between infected and uninfected mice in each T cell compartment. Pairwise cosine similarity scores were projected on the NMDS plane for each T cells compartment (sub-plots), tissue (shape), and a single mouse in different conditions (colored dots). Healthy-PBS injected mice are marked in grey dots (PBS), mice 8 days post-infection in red dots (LCMV8), and 40 days post-infection in blue dots (LCMV40). The grey ellipses on the NDMS panel of the CD4+ and CD8+ effector subplots are computed using the normal confidence ellipses. **(B)** CD4+ and CD8+ effector CDR3AAβs are highly shared between mice at day 8 and day 40 post LCMV infection. Cosine similarity was computed between effector CDR3AAβ across tissues and mice in different conditions; day 8- and 40-days post-infection (red dots), 40 days post-infection, and PBS control (blue dots), 8 days post-infection and PBS control (grey dots). All the effector sequences are in the left panel, and the effector epitope-specific clones are in the right panel. The mean is shown in black lines (n=number of paired mice cross treatments and tissues). Significant differences between mice and tissues are denoted in asterisks (p-value ****<0.0001 Kruskal-Wallis test).

To further validate whether these long-term repertoire organizational changes are driven by common TCRs, we used the same measurements described in **Figures 1B, C** to evaluate the clonal overlap in mice at different immune states (healthy vs. infected mice at day 8 vs. infected mice at day 40). Indeed, the clonal overlap was increased only in CD4+ and CD8+ effector T cells between day 8 and 40 post-infection and not in the other T cell states and between PBS and infected mice (**Figures 2A, B**). Thus, the repertoire organizational changes are driven at least in part by shared effectors TCRs detected upon infection.

### 3.3 Expansion and increased sharing in LCMV– specific TCRs

The increased sharing following infection observed in the data (**Figures 1**, **2**) did not distinguish between antigen-specific or potential bystander T cells activated by the infection. We, therefore, identified a set of antigen-specific TCRs, using tetramer purification and subsequent stringent bioinformatic filtering (see **methods** section), resulting in good reproducibility and high sequence overlap between biological replicates (**SI Figure 1F**). We used this pipeline to sort and sequence TCRs specific for 3 CD8+ and 1 CD4+ LCMV epitopes from mice at day 8 post-infection (**Figure 1A**).

A summary of the selected annotated epitope-specific TCRs is presented in **Supplemental Table 2**. A set of Herpes simplex virus CD8 specific TCRs (31) served as a control for these analyses.

**Figure 3:**
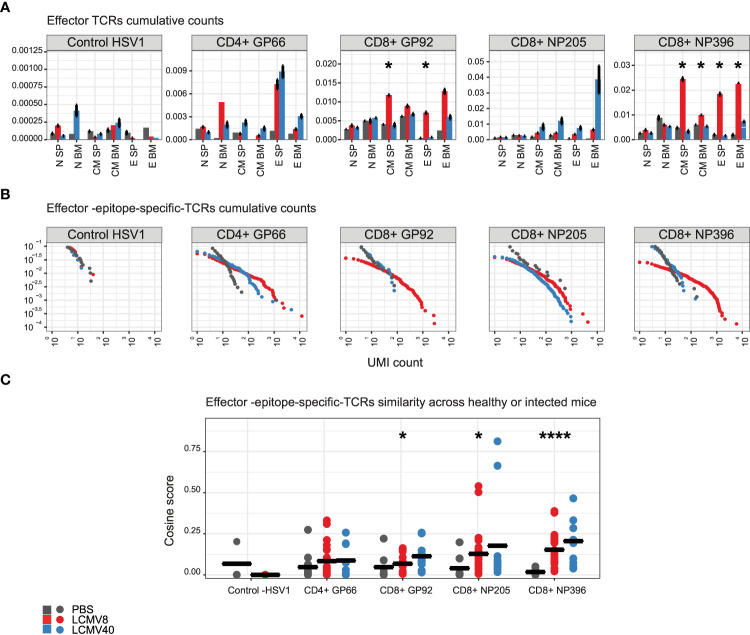
Epitope-specific CDR3AAs are mainly found in the effector state of mice after eight days of LCMV infection. The epitope-specific CDR3AAβs are annotated to healthy-PBS injected mice (grey dots and bars), mice at 8 (red dots and bars), or 40 days post LCMV infection (blue dots and bars). Each epitope-specific group is labeled above or on the X-axis. The control epitope-specific sequences are labeled “Control -HSV1”. **(A)** The mean fraction of epitope specific CDR3AAβs in each compartment, tissue, and mice condition. Error bars are SEM (n=mice number). Significant differences between mice after 8 days of infection and healthy control mice are denoted by asterisks (p-values: * <0.05, Kruskal-Wallis test). **(B)** The cumulative frequency of the effector - epitope-specific sequence. The plots show the cumulative proportion of the repertoire (y-axis) made up of TCR sequences observed once, twice, etc. (x-axis). Significant differences were obtained between 8 days post infection and PBS treated mice, in effector epitope-specific CD8+ NP396, CD8+ GP92,CD8+ NP205, and CD4+ GP66 cells (p-value=5.4e-9, p-value=6.3e-5, p-value= 1.3e-3, p-value=4.9e-6, respectively, Kolmogorov-Smirnov test). Significant differences were obtained between 40 days post infection and PBS treated mice, in effector - epitope-specific CD8+ NP205 and CD4+ GP66 cells (p-value= 5.0e-4,p-value=4.9e-6, respectively, Kolmogorov-Smirnov test). **(C)** Effector – epitope-specific sequences are highly shared across LCMV infected mice. Cosine similarity scores were calculated for each type of epitope-specific repertoire between mice and tissues. The mean is shown in black lines (n= number of paired mice and tissues). Significant differences between mice and tissues are denoted in asterisks (p-values: * <0.05, **** <0.0001 Kruskal-Wallis test).

We then looked for this set of antigen specific TCRs in the bulk repertories from the different subpopulations of T cells (**Figure 3A**). Out of the set of epitope-specific CDR3AAβs, a high fraction was found in at least one repertoire from LCMV-infected mice (**SI Table 2**, out of filtered TCRs: GP66- 43%, GP92- 65%, NP205- 92%, NP396- 64%). As expected, the maximum enrichment of the antigen-specific TCR sequences was seen in the day 8 effector and memory population. At the peak of the infection, day 8, splenic CD8+ effector and memory repertoires contained a higher fraction of NP396 and GP92 specific clones (1-2%) than NP205 clones (~0.5-0.6%), reflecting the known immunodominance hierarchy (19). We did not observe significant enrichment of CD4+ GP66 epitope-specific T cells in the CD4+ effector population. Similarly, we did not observe any significant enrichment of Herpes simplex virus type 1 (HSV1)-specific TCRs in either the effector or memory compartments. We focused on the splenic effector cells, which contained the highest fraction of epitope-specific clones and plotted the abundance profile of the annotated TCRs (**Figure 3B**). Clonal expansion, as evidenced by the presence of TCRs present at high abundance compared to unimmunized mice was observed for all epitopes and was especially pronounced at the peak of infection. No expansion of HSV1 annotated TCR sequences was observed.

We next examined sharing between the epitope specific TCR repertoires, as described in **Figure 1B** for the bulk repertoires. We observed a similar increase in repertoire similarity at day 8 post infection (**Figure 3C**) within the effector T cells for all four epitopes, although the CD4+ changes in the peak of infection were smaller and did not reach statistical significance (**Figure 3C**). Infection did not alter sharing in the control HSV1-annotated TCR set. Overall, we confirmed that infection induced a concurrent expansion and convergence of TCR sequences in effector cells, including the epitope specific repertoire.

### 3.4 Acute LCMV infection reveals patterns of CDR3 sequence sharing, mainly among the effector T cells of infected mice

**Figure 4:**
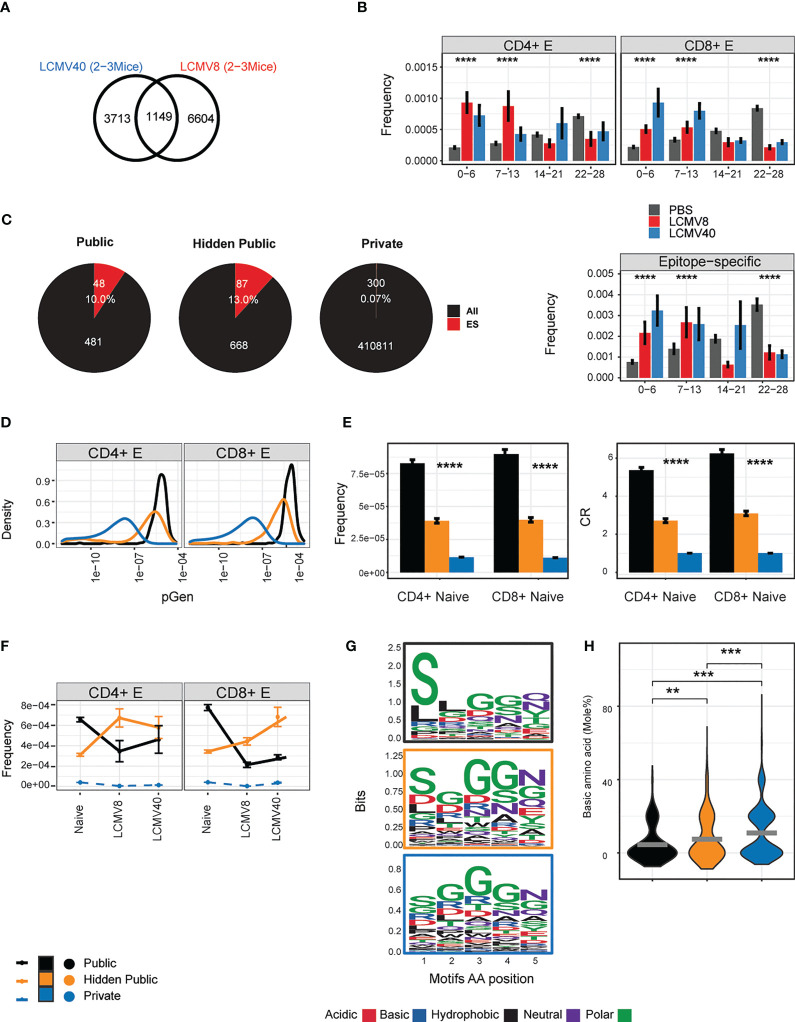
Defining properties of LCMV driven- hidden- public clones. **(A)** The number of CD4+ and CD8+ effector CDR3AAβ sequences that overlapped with most mice after 8 (LCMV8) and 40 (LCMV40) days of infection (4-9 mice, 1149,” LCMV- long-lasing TCRs”). **(B)** The sharing distribution of LCMV- long-TCRs, across the 28 mice reference cohort. The frequencies of the CD4+ or CD8+ LCMV- long-lasting CDR3βAA (1149) and the epitope-specific sequences among them (lower panel) that were undetected (0) or found in a 1-28 mice reference data set (6). Healthy-PBS injected mice are marked in grey bars (PBS), and mice 8 or 40 days post-infection are marked in red and blue bars, respectively. The frequency was calculated by normalizing the CDR3AA UMI count from each class (CD4+/CD8+) and immune state (PBS/LCMV8/LCMV40) by the total counts in all mice and tissues. Presented in the mean frequency in each sharing group (0-6,7-13,14-21,22-25). Error bars are SEM (n=sequences number). **(C)**. LCMV- long-lasing TCRs (n=1149, A) are divided into two groups according to the sharing hierarchy found in the reference data set: 1) public TCRs shared by 22-28 mice, 2) hidden public TCRs undetected (0) or found shared by 1-21 mice. CD4+ and CD8+ effector TCRs that are termed private are sequences that appeared in one mouse from the current dataset and not in the reference cohort. The total (“All”) and the epitope specific TCRs (“ES”) number and fraction are marked in white text and red color. **(D)** The probability generation (pGen) scores and CDR3AAβ for each CD4+ and CD8+ TCRs population. **(E)** CD4+ and CD8+ naïve precursor frequency and convergent recombination (CR) mean number across public, hidden, and private TCRs population. Error bars are SEM (n=sequences number). **(F)** Clonal evolution from the naïve state to 8 up to 40 days post-infection. For each TCRs population (out of 1093 TCRs) in the different immune states, points represent the mean frequency. The connected lines describe the clone time-based trajectory. Private CDR3AA in each immune state were subsampled (500) to avoid the size variation between the TCRs populations. The dashed lines represent the private population’s unique CDR3AA sequences in each immune state trajectory. **(G)** Chemical properties of the five amino acid motifs from the public, hidden public, and private (indicated by the frame colors). Significant differences were obtained between pGen distribution of hidden public TCRs and public TCRs and between hidden public TCRs and private TCRs (p-value < 2.2e-16, Kolmogorov-Smirnov test). **(H)** Each point represents a basic (H + K + R) amino acid mole percentage in each 5AA motif of the public, hidden public or private TCRs populations. The mean is shown in (n=number CDR3AAs in each group). Significant differences between public, hidden public and private TCRs are denoted in asterisks (p-values: ** < 0.01, *** <0.001, **** <0.0001 Kruskal-Wallis test).

We identified 1149 “public” CDR3 sequences which were shared between most T effector repertoires from LCMV infected mice (4-9 mice, **Figure 4A**). We hypothesized that if these TCR sequences were classical public sequences (6, 32) they would be frequently observed in repertoires of unimmunized mice. We therefore searched for these common TCRs in a repertoire database of 28 uninfected “control” mice investigated previously (6). 1093 CDR3 sequences were detected in the reference cohort and showed very variable degrees of sharing. 481 TCR sequences were shared between 22-28 mice in the reference cohort and defined as classical public TCRs. As expected, these CDR3s were enriched in the uninfected control mice in our experiment (grey bars in **Figure 4B**).

Out of the shared TCRs (1149) that were not classical public, 668 were defined as “hidden public TCRs”. Interestingly, CDR3s detected in less than 14 reference repertoires were significantly enriched in both 8- and 40-days post-infection mice (**Figure 4B**). A similar pattern was observed in the subset of the 1149 public CDR3s which were also identified as LCMV-specific by tetramer staining, although the number of such CDRs was much smaller (**Figure 4B**, lower panel). The proportion of the shared LCMV CDR3s which bound HLA-tetramer is shown in **Figure 4C**. Thus, we conclude that there is a substantial proportion of CDR3s which is highly public when comparing the effector repertoires of LCMV-infected mice but have intermediate levels of sharing in unimmunized repertoires. We refer to these as hidden public CDR3s.

The degree of sharing between repertoires in different individuals is determined in part by the probability of generating a particular TCR during somatic recombination (pGen), which can be inferred from the CDR3 sequence (25). This repertoire bias results in highly frequent naive populations encoded by many different CDR nucleotide sequences (convergent recombination degree - CR). Public CDR3s have been shown to have a much higher pGen, CR and frequencies distribution and shorter lengths than private CDR3s, explaining in part how they can be observed in many independent repertoires. We calculated these measurements for all the CDR3s shared between all LCMV-infected repertoires and stratified them according to their publicity within the control uninfected repertoires (**Figures 4D, E**). The hidden public CDR3s had pGen and length distributions which lay between that of private and public CDR3s (**Figure 4D**; **SI Figure 2B**). Hidden public CDR3s were also detected with intermediate levels of naive frequencies and CR degrees (**Figure 4E**), suggesting they hold unique repertoire bias properties, which can be fully revealed upon viral expansion. To better understand these dynamic changes, we focused on overlapped clones from effector cells of infected mice and naive cells from healthy mice. This allowed us to follow a clone- trajectory based on the average clonal frequency change from the healthy to day 8 and 40 post-infection (**Figure 4F**). While public TCRs were reduced, hidden public TCRs increased at 8 days post-infection. After 40 days of infection, the hidden public TCR changed their dynamics, CD4+ reduced, and CD8+ maintained high frequency. We note that private TCRs cannot be linked to this trajectory as they contained unique CDR3AA sequences in each mouse (**Figure 4F**, marked in blue dashed lines). However, private TCRs can represent a reference, which showed, on average, lower clonal frequency compared to the shared TCRs. Similar patterns were observed in both spleen and bone-marrow tissues, and by computing the repertoire fraction in each public, hidden public, and private populations (**SI Figure 2A**). The differences between the private, hidden public and public CDR3s were further explored via the physicochemical properties of the CDR3 amino acids.

We visualized the relative contribution of each of the central five amino acids of the CDR3, the region most likely to contact the peptide epitope (33). As shown in **Figure 4G**, serine is over-represented at the beginning of the sequence in the fully public CDR3s, while both private and hidden public sequences were more diverse (**Figure 4G**). A lower average basic amino acid was observed in the public and hidden public motifs than in the private motifs (**Figure 4H**).

### 3.5 Hidden public TCRs in the context of SARS-COV-2 infection

We hypothesized that hidden public TCRs may emerge more generally as a response to acute infection. We therefore examined the TCR repertoires of 39 individuals who tested PCR positive for SARS-COV-2 during the first wave of the pandemic in the UK (Manisty), as well as 6 individuals who remained PCR negative and seronegative throughout. As described in detail previously in (21), we identified a wave of TCRs which expanded within the first few weeks of infection in most infected individuals.

**Figure 5:**
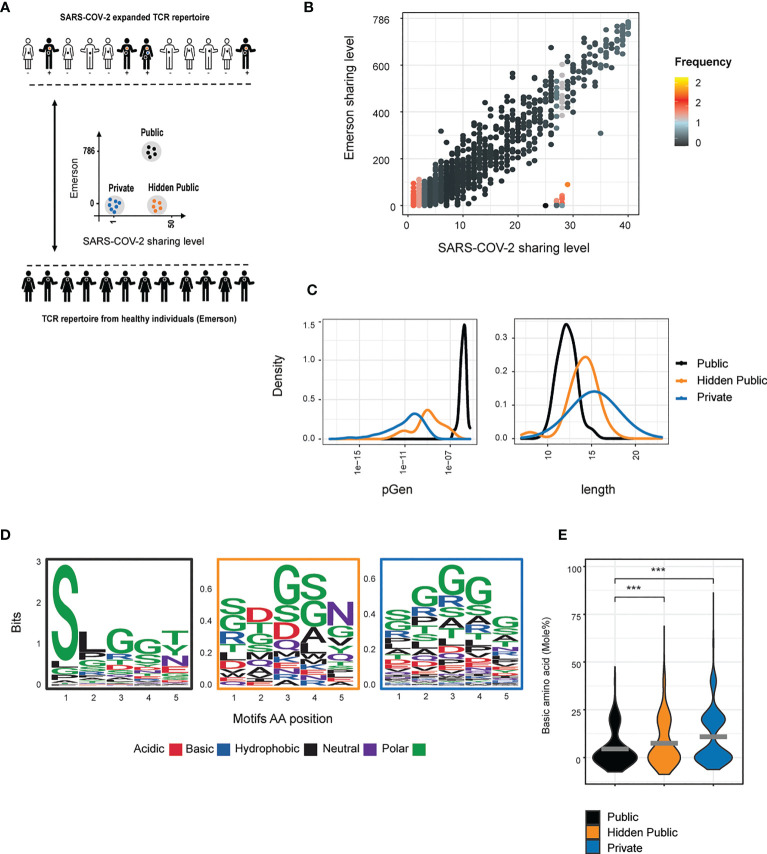
Hidden public TCRs revealed in SARS-COV-2 patients. **(A)** An overview of the collected data and analysis design **(B)** Comparison between the sharing levels of CDR3 sequences found across individuals from the Covid and the Emerson data sets. The color represents the log 10 median frequency of all CDR3AAs in each Covid sharing level (high= orange, low = black). Three TCRs populations were defined: 1) Public TCRs highly shared in both data sets (above 524 and 26 individuals in the Emerson and Covid patients, respectively, 92 CD3AAs). 2) “Hidden public” TCRs which were highly shared only among the SARS-COV-2 cohort (above 26 and below 50 individuals from the Covid and Emerson cohort, respectively, 21 CD3AAs). 3) Private TCRs exclusively detected in one patient from the Covid data set. **(C)** The probability generation scores, CDR3AAβ length distributions in reach of the defined population. **(D, E)** The chemical property of the 5 middle amino acid motifs in each of the defined populations. **(D)** Amino acid sequences logo. **(E)** Each point represents the mole percentage of basic (H + K + R) amino acid in each public, hidden public or private motifs. The mean is shown in (n=number CDR3AAs in each group). Significant differences between public, hidden public and private TCRs are denoted in asterisks (p-value *** <0.001 Kruskal-Wallis test).

To compare the level of publicity of these expanding CDR3s between the COVID-infected individuals, and uninfected individuals we utilized a reference cohort of 786 healthy individuals (34), referred to here as the Emerson data set, **Figure 5A**) collected several years prior to the SARS-COV-2 pandemic. Most of the expanded SARS-COV-2 CDR3 sequences were found in the Emerson data set (59.4%, 2794). Within the expanded set of TCRs we identified a set of classical public TCRβ sequences, which are highly shared across many healthy and SARS-COV-2 infected individuals (92 TCRs shared in more than 65% of individuals in both data sets). However, we also identified a set of CDR3 sequences that are highly shared only among the SARS-COV-2 infected individuals (21 TCRs found shared in more than 65% of SARS-COV-2 infected individuals and below 5.3% of healthy individuals) (**Figure 5B**). This set of TCRs is analogous to the hidden public TCRs from mice, which were highly shared only among LCMV infected individuals and not in the 28 reference mice. The hidden public TCRs were present at a significantly higher abundance in the repertoires of the SARS-COV-2 infected individuals (13 per million TCR) than the classical public TCRs (4 per million TCR, p-value < 2.2e-16, Wilcoxon test).

We further examined the few hidden public-TCRs which were also detected in the PCR negative individuals and found them to be present at significantly lower abundance than in the PCR positive individuals (7 CDR3AA with frequency means of 17.1 vs. 1.17 PCR positive vs. negative individuals, p-value < 2.2e-16, Wilcoxon test)(**SI Figure 2C**). The increased abundances in the PCR positive individuals support their association with antigen-driven expansion.

SARS-COV-2 driven hidden public TCR were also found in an additional higher resolution independent dataset, generated from 39 individuals prior to the SARS-COV-2 pandemic (35). This dataset has an average 2.2-fold higher number of TCRβ per individual (409519), in comparison to the Emerson data set (183211). Here as well, the SARS-COV-2 associated hidden public sequences showed intermediate abundancy, levels between public and private TCRs (**SI Figure 2D**).

The SARS-COV-2- associated hidden public CDR3s were found to have pGen and length distributions intermediate between the public and the private CDR3s (**Figure 5C**), as we observed for the LCMV hidden public sequences. Lastly, we calculated the central five amino acids usage and their average percentage of basic amino acids (**Figure 5D**). Public CDR3s showed a more constrained amino acid usage pattern than the private and hidden public CDR3s, and the public and hidden public motifs showed lower average scores of basic amino acids than the private motifs (**Figure 5E**).

Taken together, these results suggest that hidden public CDR3 sequences, with distinct properties from classical public CDR3s can be observed in different acute viral infections and host species. Thus, this phenomenon may be a generalized feature of the adaptive immune system, revealing some unexpected constraints on the diversity generated by somatic recombination in T cells.

## 4 Discussion

The well-characterized model of acute LCMV infection allowed us to probe the reactive T cell repertoire during the peak and memory phases of the viral infection. We demonstrated that viral infection drove convergent evolution in the TCR repertoire, which could be detected in both the total and the antigen-specific effector compartment. Convergence was driven by the expansion of a set of shared CDR3 sequences, which could only be detected after the antigen-specific response. These antigen-dependent shared CDR3s were seen less often than classical “public” CDR3s in unimmunized repertoires, consistent with their lower probability of generation. These observations suggest that the degree of sharing between individuals is greater than was previously thought, but that many of the shared sequences are “hidden” by being present at low abundance in the naive repertoire, and are therefore not observed in typical sampling of unimmunized mice, which sequence only a tiny proportion of the total repertoire. Strikingly, hidden public TCRs were also identified in SARS-COV-2 infected individuals, supporting the notion that these findings represent a broader and conserved phenomenon.

We examined in greater detail a subset of shared LCMV-dependent effector T cells which persisted in the repertoire until at least day 40 post-immunization. We searched for these TCRs in an independent cohort of 28 antigen-naïve mice (6). These persistent shared CDR3s were found in zero to six of these control repertoires, defining a new intermediate level of publicity. We hypothesize that the “hidden public” TCRs originate from naive cells which are generated at a sufficiently high frequency to be present in many naive repertoires, but are present at low abundance in the naive repertoire, resulting in them not being detected in routine TCR sampling. However, following infection, T cells expressing these shared CDR3 consistently expand and differentiate into effector cells as a result of exposure to LCMV peptides. As a consequence, their abundance reaches a critical level at which they are consistently detected in the repertoire samples we analyze. Consistent with this hypothesis, we find that the “hidden public” CDR3s have higher naive precursor frequencies, more convergent recombination, and higher generation probabilities than random sets of CDR3s (which are mostly private to a single mouse and compartment). However, they have lower levels of these metrics than classical “public” CDR sequences.

The differences between the public and “hidden public” CDR3s may reflect different functional properties. Indeed, while public TCRs were shown to be more self-immunity-associated (6), the hidden public TCRs react to viral infections. Although the mechanisms remain incompletely understood, increasing levels of naive precursor T cell frequencies have been shown to drive more significant peptide MHC responding capacities (19, 31). The range of naive precursor frequencies and the phenotype heterogeneity (36) has yet to be fully determined but might explain the hidden public pre-exposure antigenic preferences.

The hidden public TCRs appear to be a broader phenomenon found also in other viral infections and species. The first SARS-COV-2 pandemic wave offered a good model for a primary viral infection in humans. We searched the expanded SARS-COV-2 TCRs (37) in a large cohort of healthy humans, and detected a set of TCRs that were highly shared across SARS-COV-2 infected individuals but showed less publicity in a cohort of pre-pandemic TCR repertoires. The detection of sharing even in the genetically heterogeneous HLA-diverse human setting is interesting, and will merit further study. Similar to the LCMV hidden public population, these TCRs had intermediate generation probabilities.

We investigated whether the “hidden public” CDR3s also showed distinct amino acid composition, which might explain their more frequent selection in the thymus (38) or their higher abundance in the naive repertoires. Since these hidden public TCRs originated from a diverse set of HLA genotype, we focused on the five amino acid middle of the CDR3AA, a region associated with binding the peptide within the MHC complex (33). The Covid and LCMV hidden public motifs showed higher amino acid diversity than the public motifs. In addition, we found that public and hidden public motifs tend to include less positively charged amino acids compared to private motifs, suggesting they hold conserved binding properties. We can speculate that the hidden public amino acid constraints might provide an evolutionary cross-reactive advantage, allowing them to react to foreign and self-antigens (39). However, further study is required to better understand the developmental process, driving the generation preference of the hidden public TCRs.

The study we present here has several limitations. The number of individuals analyzed and epitope-specific sequences were relatively small, limiting the amount of robust statistical analysis that could be carried out. Another limitation is that the analysis of the post-infection repertoires was limited to two time points. We also recognize that the effector functional state we defined was based on a rather simplistic and limited panel of cell surface markers, which could result in heterogeneous effector memory phenotypic states, especially at late post-infection time. In addition, the bulk TCRβ chain analysis cannot capture the absolute clonal identity which comprises paired α and β chains. The TCR α chain is less diverse and can be expressed twice (Dual TCRα) in virus-specific CD4+ and CD8+ T cells during acute responses (up to 60%) (40), highlighting the complexity of using the TCRα chain as a clone identifier.

This study describes a naive precursor population carrying a shared set of CDR3s capable of providing a rapid response to viral infections. We coin the term “hidden public” to describe this population. Our results suggest that the TCR repertoire may be more constrained, and hence more similar between individuals, than current dogma supposes. Deeper understanding of the processes which shape this repertoire, and determine the level of inter-individual sharing is important for understanding the antiviral response and in rational design of next-generation vaccines.

## Data availability statement

The datasets presented in this study can be found in online repositories. The names of the repository/repositories and accession number(s) can be found below: https://www.ncbi.nlm.nih.gov/sra/PRJNA954849.

## Ethics statement

The animal study was reviewed and approved by Council for experiments on animals (IACUC) Weizmann institute. Written informed consent was not obtained from the individual(s) for the publication of any potentially identifiable images or data included in this article.

## Author contributions

MM designed the study, prepared and analyzed the data, and wrote the manuscript. SR-Z: 1. AB: 2. BC: 3. NF: 1,3. AM: 1,3. Contributed with: 1. Design and conception of the study 2. Experimental preparation of the data 3. Data analysis 4. Writing the manuscript. All authors contributed to the article and approved the submitted version.

## In memoriam

Sadly, NF died after a long battle with illness before the study could be completed. We have attempted to complete this study in the spirit in which it was undertaken but acknowledge that we raised more questions than answered. We dedicate this study to his memory. We greatly appreciate that the Armstrong LCMV viral strain was a kind gift from Prof. Matteo Iannacone (San Raffaele Institute).

## Supplementary material

Supplementary Figure 1
**(A)** Representative sorting gates of CD4+ cells from one mouse in each condition (PBS/LCMV8/LCMV40). **(B)** Representative sorting gates from CD8+ T cells specific for NP205 peptide. From the CD8+ population, 2.86% are positive for the MHC class I NP205 tetramer (lower right panel) and almost all CD4+ cells are negative (0.018%, lower left panel). The CD8+ cells that are negative for the tetramer were also sorted and analyzed. **(C)** The number of UMIs correlates with the sorted cell number. Dots correspond to the sum of UMI count versus the sorted cell number in mice 8 days post LCMV infection. **(D)** Cumulative frequency distributions in CD8+ and CD4+ naive central memory and effector repertoires from spleen and bone marrow. Healthy control, and mice after 8- or 40-days of infection are marked in colored dots (gray, red and blue dots, respectively). Significant differences were obtained between day 8 post-infection and PBS treated mice, in the bone-marrow CD8+ and CD4+ effectors (p-value < 2.2e-16, p-value=3.1e-6, respectively, Kolmogorov-Smirnov test) and in the following splenic compartments: CD8+ central memory CD8+ effector, CD4+ effector (p-value < 2.2e-16, Kolmogorov-Smirnov test). Significant differences were obtained between day 40 post-infection and PBS treated mice, in splenic and bone-marrow CD4+ effector (p-value =1.3e-9 and 2.2e-11, respectively, Kolmogorov-Smirnov test) and bone-marrow CD8+ central memory and CD8+ effector (p-value =3.7e-11 and 8.9e-4, Kolmogorov-Smirnov test). **(E)** The Renyi diversities of order 0, 0.25, 0.5, 1, 2, 4 Renyi values were computed from sequence frequencies at equal sizes (1000 in the spleen and 100 in the bone marrow), averaging values over 100 repeated samplings. Each color represents one CD4 + or CD8+ compartment from one mouse in a single condition. See legend for symbols and color code. **(F)** NP396 epitope-specific clones from two biological repetitions are positively correlated in the obtained UMI counts. Each point is the UMI count of a single CDR3AAβ found in the two repetitions (Rep1/Rep2).

Supplementary Figure 2
**(A)** Similar frequencies in spleen and bone marrow tissues of a single mouse, immune state (PBS/LCMV8/LCMV40), CD4+ or CD8+ class, and TCRs population. Frequencies are calculated by the sum of UMI counts per TCRs population (public/hidden public/private) divided by the total UMI count sum in each mouse, immune state, tissue, and T cell class. R2 coefficient scores are marked in each subplot. **(B)** CDR3AAβ length distributions in reach of the defined population. **(C)** The frequency of hidden public TCRs found in healthy individuals (7 CDR3AAs, PCR negative) and SARS-COV-2 infected individuals (PCR positive). Mean values are marked in black lines. Significant differences are marked in p value (Wilcoxon test). (D)SARS-COV-2 –associated hidden public TCRs detected in high resolution pre- SARS-COV-2 pandemic dataset. The three-populations identified SARS-COV-2 individuals were searched in the Britanova et al. data set (35). Each bar represents the mean frequency of SARS-COV-2 associated- public TCRs (all 92 detected, black bar), or hidden public TCRs (7 out of 21 detected, orange bar) and private TCRs (blue bars). Error bars are SEM (n=sequences number). Significant differences between public, hidden public and private TCRs are denoted in asterisks (p-values: *< 0.05, *** <0.001, Kruskal-Wallis test).

Supplementary Table 1 Number of CDR3NT and CDR3AA sequences, and sum of UMI counts for each T cell state (“ClassState”), tissue (“Tissue”), and mouse number (“Mice”) in healthy (“PBS”) or infected conditions (8 or 40 days post- LCMV infection, LCMV8 and LCMV40, respectively).

Supplementary Table 2 Number of CDR3AA sequences and sum of UMIs for each epitope-specific type during the filtering process. The sequences were obtained from the tetramer isolation experiment (see Methods section, “All” column). Epitope-specific TCRs were filtered based on: 1) top 1,000 sequences, and 2) absence in the unbinding-tetramer populations and across multiple epitope-specific types (“Filtered”). The number of filtered epitope-specific TCRs found in the bulk samples (LCMV40, LCMV8, PBS, and multiple T cell states) is presented in the “Found in bulk” column.
