# Supplementary figures and images for: Viral infection reveals hidden sharing of TCR CDR3 sequences between individuals
Source: Front Immunol. 2023 May 30;14:1199064. doi: 10.3389/fimmu.2023.1199064 (PMC10266217; doi:10.3389/fimmu.2023.1199064)

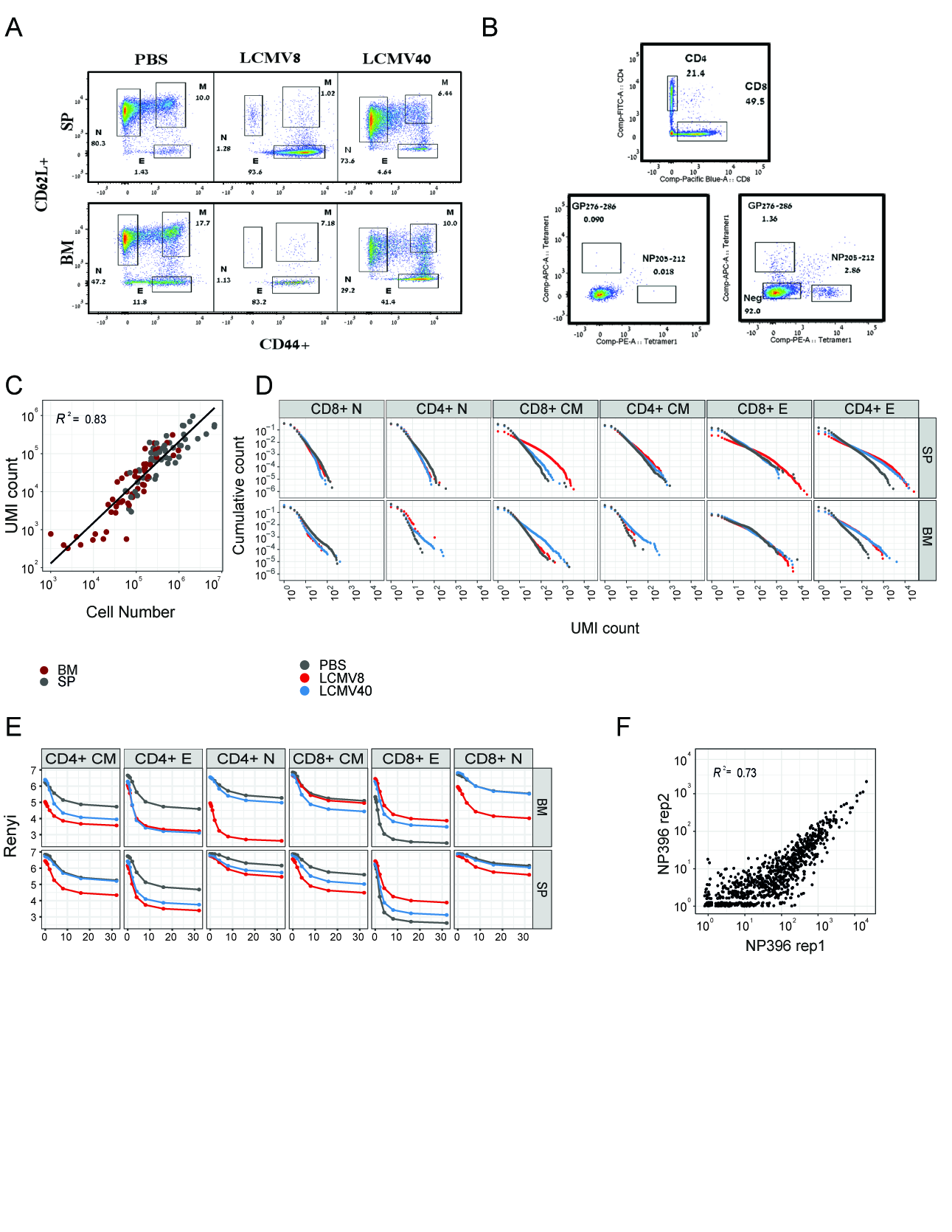

Supplement: Supplementary Figure 1 — (A) Representative sorting gates of CD4+ cells from one mouse in each condition (PBS/LCMV8/LCMV40). (B) Representative sorting gates from CD8+ T cells specific for NP205 peptide. From the CD8+ population, 2.86% are positive for the MHC class I NP205 tetramer (lower right panel) and almost all CD4+ cells are negative (0.018%, lower left panel). The CD8+ cells that are negative for the tetramer were also sorted and analyzed. (C) The number of UMIs correlates with the sorted cell number. Dots correspond to the sum of UMI count versus the sorted cell number in mice 8 days post LCMV infection. (D) Cumulative frequency distributions in CD8+ and CD4+ naive central memory and effector repertoires from spleen and bone marrow. Healthy control, and mice after 8- or 40-days of infection are marked in colored dots (gray, red and blue dots, respectively). Significant differences were obtained between day 8 post-infection and PBS treated mice, in the bone-marrow CD8+ and CD4+ effectors (p-value < 2.2e-16, p-value=3.1e-6, respectively, Kolmogorov-Smirnov test) and in the following splenic compartments: CD8+ central memory CD8+ effector, CD4+ effector (p-value < 2.2e-16, Kolmogorov-Smirnov test). Significant differences were obtained between day 40 post-infection and PBS treated mice, in splenic and bone-marrow CD4+ effector (p-value =1.3e-9 and 2.2e-11, respectively, Kolmogorov-Smirnov test) and bone-marrow CD8+ central memory and CD8+ effector (p-value =3.7e-11 and 8.9e-4, Kolmogorov-Smirnov test). (E) The Renyi diversities of order 0, 0.25, 0.5, 1, 2, 4 Renyi values were computed from sequence frequencies at equal sizes (1000 in the spleen and 100 in the bone marrow), averaging values over 100 repeated samplings. Each color represents one CD4 + or CD8+ compartment from one mouse in a single condition. See legend for symbols and color code. (F) NP396 epitope-specific clones from two biological repetitions are positively correlated in the obtained UMI counts. Each point is the UMI [file Image_1.tif]

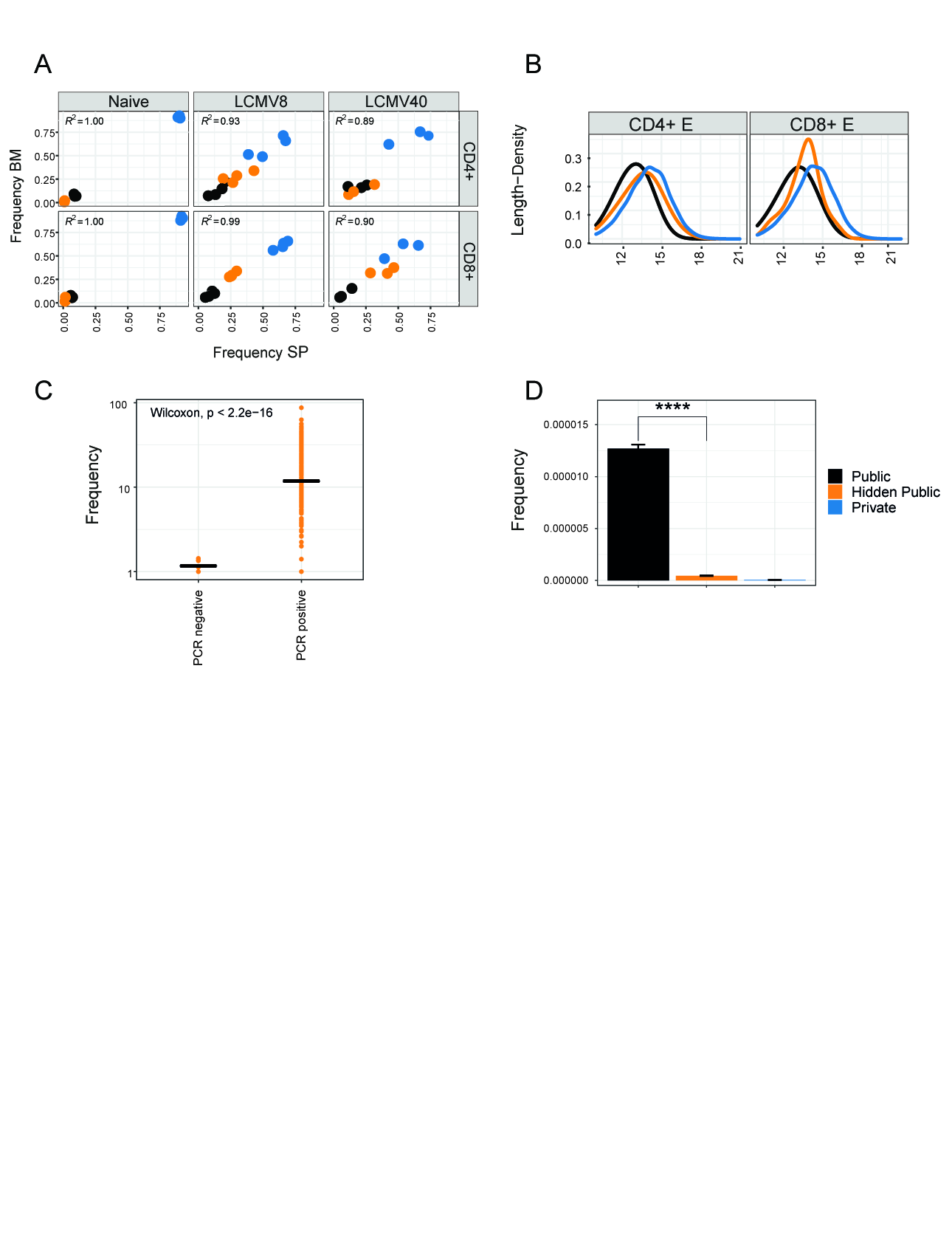

Supplement: Supplementary Figure 2 — (A) Similar frequencies in spleen and bone marrow tissues of a single mouse, immune state (PBS/LCMV8/LCMV40), CD4+ or CD8+ class, and TCRs population. Frequencies are calculated by the sum of UMI counts per TCRs population (public/hidden public/private) divided by the total UMI count sum in each mouse, immune state, tissue, and T cell class. R2 coefficient scores are marked in each subplot. (B) CDR3AAβ length distributions in reach of the defined population. (C) The frequency of hidden public TCRs found in healthy individuals (7 CDR3AAs, PCR negative) and SARS-COV-2 infected individuals (PCR positive). Mean values are marked in black lines. Significant differences are marked in p value (Wilcoxon test). (D)SARS-COV-2 –associated hidden public TCRs detected in high resolution pre- SARS-COV-2 pandemic dataset. The three-populations identified SARS-COV-2 individuals were searched in the Britanova et al. data set (35). Each bar represents the mean frequency of SARS-COV-2 associated- public TCRs (all 92 detected, black bar), or hidden public TCRs (7 out of 21 detected, orange bar) and private TCRs (blue bars). Error bars are SEM (n=sequences number). Significant differences between public, hidden public and private TCRs are denoted in asterisks (p-values: *< 0.05, *** <0.001, Kruskal-Wallis test). [file Image_2.tif]
